# Learning of a simple grapho-motor task by young children and adults: similar acquisition but age-dependent retention

**DOI:** 10.3389/fpsyg.2015.00225

**Published:** 2015-03-05

**Authors:** Mona S. Julius, Esther Adi-Japha

**Affiliations:** ^1^School of Education, Bar-Ilan UniversityRamat Gan, Israel; ^2^Gonda (Goldschmied) Multidisciplinary Brain Research Center, Bar-Ilan UniversityRamat Gan, Israel

**Keywords:** skill learning, developmental invariance hypothesis, kindergarten, motor skills, procedural memory

## Abstract

Many new skills are acquired during early childhood. Typical laboratory skill learning tasks are not applicable for developmental studies that involve children younger than 8 years of age. It is not clear whether young children and adults share a basic underlying skill learning mechanism. In the present study, the learning and retention of a simple grapho-motor pattern were studied in three age groups: 5–6, 7–8, and 19–29 years. Each block of the task consists of identical patterns arranged in a spaced writing array. Progression across the block involves on-page movements while producing the pattern, and off-page movements between patterns. The participants practiced the production of the pattern using a digitizing tablet and were tested at 24 h and 2 weeks post-practice. All age groups produced the task blocks more quickly with practice, and the learning rate was inversely related to the initial production time. All groups exhibited additional gains 24 h post-practice that were well-retained 2 weeks later. The accuracy of the participants was maintained throughout the 2-weeks period. These findings suggest that young children and young adults use a similar mechanism when learning the task. Nevertheless, the 6-years-old spent more time off-page during retention testing than when tested at 24 h post-practice, thus supporting the notion that an age advantage may exists in the long-term retention of skills due to planning-dependent aspects.

## INTRODUCTION

A central neurobehavioral tenet asserts that the long-term retention of memories is subserved by two separate and distinct systems: a declarative system which retains singular experiences and memories of facts and events, and a procedural system which addresses repeated experiences and memories of skills and habits (Cohen and Squire, 1980; Brown and Robertson, 2007). Declarative and procedural memory processes interact closely during learning in everyday life. Procedural memory plays a major role during childhood when many new motor skills are acquired.

The cognitive processes and neural substrates that mediate our capacity to acquire and retain new skills have been studied extensively in recent years by following the time-dependent course of learning (for reviews see Robertson et al., 2004; Doyon and Benali, 2005; Censor et al., 2012). An extensive body of research has shown that in adults, the development of skilled performance often extends beyond the actual training experience. This has recently been shown to occur also in children (Dorfberger et al., 2007; Savion-Lemieux et al., 2009). Training-dependent gains in performance may appear hours after the termination of training, for example 24 h post-training. It has been proposed that these delayed (“oﬄine”) gains in performance reflect memory consolidation neural processes within the processing stream that are involved in task performance, i.e., these processes are triggered by the training experience but require time to reach completion (Feldman, 2009; Xu et al., 2009; Caroni et al., 2012). The resulting gains are maintained for weeks (e.g., Korman et al., 2003; Dorfberger et al., 2007; but see Savion-Lemieux and Penhune, 2005).

In the previous decade, attention was devoted to the developmental difference that occurs from kindergarten to adulthood in gains accrued during a training session (Vinter and Perruchet, 2000; Ferrel-Chapus et al., 2002; Janacsek et al., 2012; Witt et al., 2013; Hodel et al., 2014). However, less attention was devoted to post-training processes in young children (Vinter and Perruchet, 2000; Wilhelm et al., 2008; Savion-Lemieux et al., 2009). It is not clear whether the generation of long-term procedural memory (i.e., the consolidation and retention phases) is similar among kindergarteners, older children, and young adults. Accumulating evidence supports the notion of faster memory consolidation during wakefulness in children (Dorfberger et al., 2007; Wilhelm et al., 2008; Ashtamker and Karni, 2013; Adi-Japha et al., 2014). However, it is unclear whether age-dependent differences emerge during retention. Furthermore, despite its importance, the retention of skills has not been studied in children younger than 8 years of age.

There exist several standardized tests of declarative memory for preschool children (e.g., within the Kaufman Assessment Battery for Children (K-ABC-II; Kaufman and Kaufman, 2004) and the children’s memory scale (Cohen, 1997, see also Adi-Japha, 2013). However, there are no standardized tests that assess procedural memory. One reason may be that available tasks for testing skill learning in young children are usually adaptations of grapho-motor (Vinter and Perruchet, 2000; Ferrel-Chapus et al., 2002), or motor-sequencing (Wilhelm et al., 2008; Savion-Lemieux et al., 2009; Janacsek et al., 2012; Hodel et al., 2014) tasks. These tasks generally require extensive training, and the learning activities involved can be very different from activities that young children learn to master. Task-specific accuracy demands and use of explicit strategies impede developmental studies of skill acquisition both within (Janacsek et al., 2012; Witt et al., 2013) and between (Wilhelm et al., 2008; Savion-Lemieux et al., 2009) training sessions. Young children may fail to improve in more complex tasks (e.g., Huyck and Wright, 2011; Vasudevan et al., 2011), or their results may depend on broader constructs, such as attention and task motivation (Hodel et al., 2014).

Furthermore, older children and young adults may reach a performance ceiling for accuracy (Vinter and Perruchet, 2000; Savion-Lemieux et al., 2009) or a plateau for speed (Wilhelm et al., 2008) during the training session, whereas young children may improve continuously. These performance differences at the end of the training session may further complicate the comparison of consolidation and retention processes across age groups. Children may exhibit consolidation gains in accuracy 24 h post-training in tasks in which adults exhibit a performance ceiling for accuracy during training (Savion-Lemieux et al., 2009). When a performance plateau for speed is observed only in adults, it is difficult to interpret differences in consolidation gains because these differences are related to whether the plateau was reached by the end of the training session (Hauptmann et al., 2005).

A grapho-motor learning task, suitable for kindergarten children, was recently introduced (Adi-Japha et al., 2011). The task requires the reproduction of a novel pattern during training, 24 h post-training and 2-weeks post-training. On each block of this task, the participants are asked to repeatedly connect three dots to form an “invented letter.” Identical patterns are arranged in a spaced writing array. The participant connects the dots that form the first pattern, and then connects the dots that form the following pattern, until the end of the block. Progression across the block follows the writing direction, and is composed of movement on the page when the participant is producing the pattern, and movement off-page (in the air) between patterns. The processes of forming the patterns across a block resemble handwriting production. The task is typical of kindergarteners’ activities and was designed to minimize accuracy demands. The skill learned in this task is the production of the pattern. The learning protocol of the skill confirms with the definition of procedural learning that leads to a formation of long-term memory for the learned skill (procedural memory, dealing with memories for skills). The individual is aware of acquiring the skill (defined as explicit procedural learning, Robertson et al., 2004). The to-be-learned pattern resembles shapes commonly found in developmental studies of visual-motor skills (e.g., Beery et al., 1997; Meisels et al., 1997). Copying such shapes in kindergarten was found to predict academic achievement later in school (Grissmer et al., 2010; Cameron et al., 2012).

The invented letter task (ILT) was introduced in order to study procedural learning in kindergarteners with language impairment and in typically developing peers who had comparable visual-motor integration skills (Adi-Japha et al., 2011). The findings indicated that both groups maintained a low error rate throughout the 2-weeks period. The children’s speed (i.e., the time taken to complete a block) improved during training, but only the typically developing kindergarteners further increased their speed at 24 h post-training. Their performance level was retained 2 weeks post-training.

The aim of the current study was to compare the learning, consolidation, and retention of the grapho-motor pattern (i.e., the ILT) in kindergarteners, second graders and young adults. Based on the previous findings in kindergarteners (Adi-Japha et al., 2011), we hypothesized that in addition to learning during the training phase, all three age groups would exhibit consolidation gains and retention of this simple task in terms of overall speed, while maintaining accuracy. Specifically, we hypothesized that improvement in speed would not come at the expense of accuracy (no speed-accuracy tradeoff). Gradual improvement in task performance across multiple sessions without speed-accuracy tradeoff is recognized as a characteristic of skill acquisition (procedural learning) in both motor and perceptual domains (Censor et al., 2012). Such learning of the task across the three age groups would suggest that the procedural learning processes that underlie learning in adults are present in early childhood, and can be tested using the ILT. This would enable future developmental studies of different factors associated with skill learning.

### RESEARCH HYPOTHESES

In line with previous studies of motor skill learning, we expect movement to be faster and more accurate with age (e.g., Dorfberger et al., 2007; Adi-Japha et al., 2014).

The current study focuses on learning, and therefore improvement of production measures between successive time points, in the three age groups, is of interest.

#### Training phase

We expect improvement during training in all age groups. However, because children’s baseline is poorer than that of adults we expect a higher improvement rate during training in children.

Due to task simplicity, and based on the previous study in kindergarten children (Adi-Japha et al., 2011) we expect that by the end of training, performance improvement in children would be moderate or even reach a plateau. We assume that adults would demonstrate a similar level of improvement across the last training blocks.

#### Consolidation phase

A low rate of improvement, or a plateau, reached by the end of the training session, suggests that all age groups would demonstrate consolidation gains (Hauptmann et al., 2005). Following a previous study in older children and adults we assume that a similar level of gains would be accrued 24 h post-training by the three age groups (Dorfberger et al., 2007).

#### Retention

Based on the previous study in kindergarten children (Adi-Japha et al., 2011) that showed that typically developing kindergarteners were able to retain consolidation gains for 2 weeks, we assume retention of gains in the three age groups.

The analyses of handwriting production of less skilled vs. skilled writers, or of children vs. adults, suggests two major differences: less skilled writers have longer durations between writing units and they use more segments to produce writing units (Rosenblum et al., 2006). Adults and more advanced writers are able to plan the writing of the next unit while executing the previous one, whereas children and less skilled writers rely more on the off-page time (in the air, pause-time) between the units, for planning. Reliance on off-page time for higher-order processes such as planning causes difficulties in handwriting to be reflected in larger off-page time (Rosenblum et al., 2003, 2006; Sumner et al., 2014). The use of additional segments by less skilled writers is interpreted as less fluent production (Adi-Japha et al., 2007). Skilled writers are able to pre-plan curved trajectories, while less skilled writers segment their movements in order to conform to accuracy demands (Sosnik et al., 2004, 2007).

Previous studies of procedural memory in children and adults suggest that long-term retention is more susceptible to retrieval of explicit aspects of the task that are related to planning and declarative knowledge (Savion-Lemieux and Penhune, 2005; Adi-Japha and Abu-Asba, 2014). A decrease with time in performance level characterizes movements composed of discrete units, rather than continuous ones. This decrease in performance level increases with the length of retention interval (for a review see Schendel et al., 1978). We therefore expected larger age differences at retention testing than during training or 24 h post-training, in off-page time and the use of more segments (Savion-Lemieux and Penhune, 2005), thereby differentiating retention from learning.

## MATERIALS AND METHODS

### PARTICIPNATS

Seventy-six participants were recruited for this study. The study included 36 5- to 6-years-old kindergarteners (*M* = 74 months, SD = 3.85 months, range 67–80 months; 18 girls), 20 7- to 8-years-old second graders (*M* = 96 months, SD = 5.18 months, range 90–107 months; 10 girls), and 20 young adults (*M* = 24.5 years, SD = 1.5 years, range 19–29 years; nine females). The participants were recruited from centrally located areas with a medium to high socioeconomic status. Approval was obtained from the Ministry of Education (10.32/235/2010, 10.32/514/2011), and the parents of the children signed the Ministry of Education’s consent forms. All participants were right-handed based on the Hand Dominance Questionnaire (Oldfield, 1971; kindergarten *M* = 0.88, SD = 0.11; second-grade *M* = 0.88, SD = 0.10; adults *M* = 0.86, SD = 0.08). The parents answered this questionnaire for the kindergarteners and the second graders.

### MEASURES

In addition to the study task, the kindergarteners and second graders were administered two sequential short-term memory tests: the Number Recall test and the Hand Movement test from the Sequential subtest of the K-ABC (K-ABC, Kaufman and Kaufman, 1983). This version of the K-ABC was adapted for Hebrew and has been normalized in Israel (Phizer et al., 1995).

#### Number Recall test

The experimenter read aloud a random string of numbers between 2 and 7 digits in length. The children were asked to repeat the string of numbers in the same order. The testing continued until the child made three consecutive errors.

#### Hand Movement test

The children were presented with a random sequence of hand movements (made with the fist, palm or side of the hand) of varying lengths (between 2 and 5 movements) and were asked to imitate the movements. The testing continued until the child made three consecutive errors. The normalized scores of these two tests were correlated in kindergartners and second graders, *r*(36) = 0.38, *p* < 0.02 and *r*(20) = 0.56, *p* < 0.01, respectively.

#### The study task

The ILT (Adi-Japha et al., 2011) was used to study the time-dependent course of motor skill acquisition. The task consists of point-to-point planar movements and does not require a memory load because the visual stimuli and the direction of movement are available to the participants throughout the task. The difficulty of this task was adjusted to letter learning tasks previously used in kindergarteners (e.g., Longcamp et al., 2005).

**FIGURE 1:**
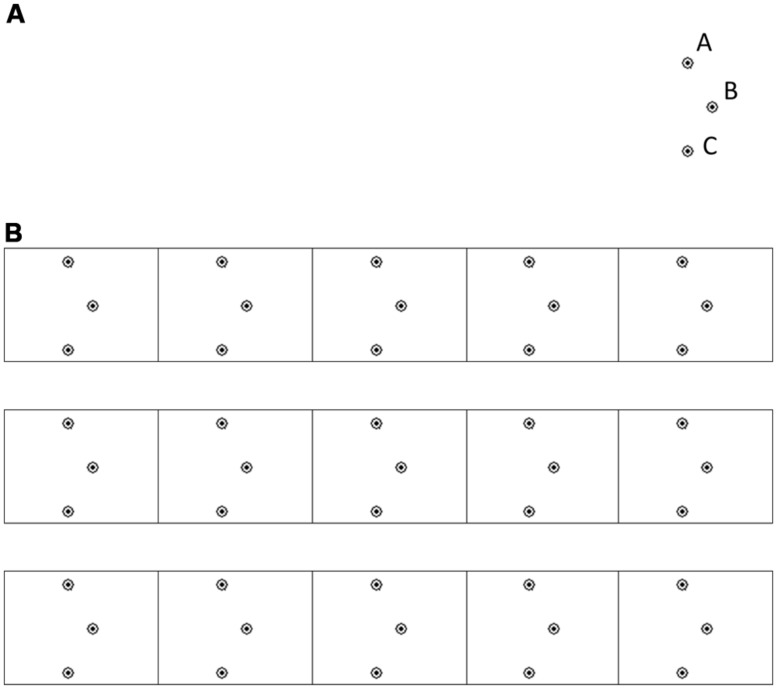
**The “invented letter” stimuli. (A)** A single stimulus. Writing direction A-B-C. **(B)** A block of the invented letter task. The writing direction is from right-to-left.

In the ILT, the children are asked to connect three circled dots using lines (**Figure 1A**: A→B→C, segment length 1.2 cm, circle outer diameter 3 mm, shape width 6 mm) to form an invented letter. Movement progression within a block was from right to left (as in Hebrew writing). Each experimental block was comprised of three rows of five dot-to-dot shapes (**Figure 1B**).

*Apparatus.* The ILT was recorded using a digitizing tablet (WACOM Intuos 2, 200 Hz sampling rate, nominal accuracy of 0.02 mm). The participants performed the task on half of an A4-sized paper, which was firmly attached on top of the digitizing tablet. One block of the task (**Figure 1B**) was printed on the paper. Participants produced the task using an ink stylus which resembles a ballpoint pen and leaves a visible ink trace on the page (i.e., the participants were able to see their drawings on the page). The pen digitally records the drawing movements (place with respect to tablet surface, and pressure) and the data is transmitted to the computer. After completion of each block the piece of paper was replaced.

*Coding.* The writing product was evaluated using a MATLAB computer program which was designed for this particular task. The program computed performance time and accuracy.

Production time was computed from the first touch of the pen tip on the page until task completion. The time it took the participant to produce the block was divided into on-page time (i.e., the total time that the pen tip touched the paper) and off-page time. The digitizing tablet provided a flag measure of the on-page or off-page contact, which was calibrated by the axial pressure of the writing stylus on the tablet surface. The on-page time was measured directly. The off-page time was computed as the difference between the overall time and the on-page time.

Erroneous shapes included shapes that were *not* produced in one continuous movement (e.g., a shape that was composed of two segments) or shapes that were too narrow or wide with respect to the midpoint of the shape (point “B”, **Figure 1A**). The midpoint was located 6 mm to the right of the upper point (“A”) on the *x*-axis. Shapes were considered erroneous if they were less than 0.325 mm or more than 0.875 mm in width. These limits visually correspond to an A-B-C line (**Figure 1A**) outside the “B” circled area, and take into account the thickness of the drawn line. The distance of the curve from the point “B” was also assessed, as an additional spatial measure. The on/off flag measure was used to evaluate the number of segments utilized to produce each shape.

An independent rater evaluated the accuracy via visual inspection of the drawings in order to test the reliability of the error measure. The rater evaluated the four initial blocks and the four final blocks of 54 randomly selected participants (70% of the participants, 28% of the dataset). The overall correlation was high, *r* = 0.70, *p* < 0.001. Differences emerged because the digitizing tablet was more sensitive to pen lifts (which are not always visible) and less sensitive to the exact position of the line with respect to the circled dot area.

*Analyses.* Skill acquisition was evaluated following Adi-Japha et al. (2011), where the average across four blocks was used as the measure of a particular time point. Averaging across blocks to evaluate performance is common in studies of skill learning (e.g., Dorfberger et al., 2007; Wilhelm et al., 2008). The following four testing points that spanned the 2-weeks period were used in the analyses: (a) initial training (blocks 1–4 on day 1), (b) end-training (blocks 9–12 on day 1), (c) 24 h post-training (four blocks, assessed on day 2, 24 h post-training), and (d) 2 weeks post-training (four blocks, assessed 2 weeks after day 1). The four time points were subjected to a repeated measures analysis of variance (ANOVA). Follow-up analyses pertained to three phases: training, consolidation (24 h post-training vs. end-training performance) and retention (2 weeks-post training vs. 24 h post-training performance). A Greenhouse–Geisser correction was used when appropriate. A Scheffé (1959) correction was used for multiple comparisons.

Two additional measures commonly used for the evaluation of learning curves were adopted in the analysis of the production times: the slope of improvement across four blocks that constitute a time point, and a power-law analysis. The slope of improvement was individually evaluated as the linear regression coefficient fitted to the four blocks. Power-law functions have been shown to robustly fit the group-averaged learning curves (e.g., Rickard, 2007). In the power-law analysis, a power-law function was fitted to the group average performance across the 12 training blocks and extrapolated to an additional four blocks. The difference between the group-averaged extrapolation and individual actual performance at 24 h post-training was used as a stringent test of whether or not consolidation gains exist (Rickard, 2007; Adi-Japha et al., 2014).

### PROCEDURE

The experimenter met individually with each participant on three occasions. Training was conducted on day 1. Consolidation was tested 24 h post-training (day 2), and the retention session occurred 2 weeks post-training. After retention testing, children performed the Number Recall test and the Hand Movement test.

The experimenter introduced the task to the participants on each of the three experimental days. She told the participants that the line they draw should go through the three encircled dots using one continuous movement. The experimenter then told the participants that they should start each row on the right-hand side of the page and progress from right to left (e.g., “Start each line here,” the experimenter pointed where to start, “and continue along here”). The experimenter *did not* demonstrate how to produce the task.

All participants were given one practice block in a paced manner at the beginning of each experimental day. Participants who either did not succeed in connecting the dots of the five patterns in the first row or who did not start on the right-hand side of the page were given an additional explanation with emphasis on accurately progressing between the shapes and lines before they produced the second row. All adults completed the practice with almost no errors. Children, and especially kindergarteners, erred on their initial practice trial. If a child did not go through the encircled points, the experimenter demonstrated how to go through the encircled area on one particular shape. If a child did not begin the row on the right-hand side, s/he was corrected. The children did not begin the experiment before correctly producing at least one row and moving correctly to the next row. Following the practice procedure, the participants were given the first block and were asked to connect the dots as rapidly and accurately as possible (e.g., “Connect the dots as quickly and accurately as you can in one stroke without stopping, until you complete all of the shapes on the page”).

Overall, 20 identical blocks of the task were performed. On all testing days, the blocks were separated by 15–30 s. After completion of each experimental block, the experimenter attached an identical sheet of paper to the digitizing tablet for completion of the next block. No feedback was provided on any performance measure. Only general encouragement was provided (e.g., “You are doing fine,” “Pay attention to the task,” and “Remember to be as quick and accurate as possible!”). The children were corrected if they started a row on the left-hand side.

The training session took ∼15, 20, and 25 min for the adults, second graders and kindergarteners, respectively. This session included the initial training procedure. The other two sessions took up to 5 min. Younger children were typically happy to participate in the study, but needed more encouragement toward the end of each session. The prizes, which consisted of school supplies (e.g., markers and stickers), were distributed at the end of each session.

## RESULTS

Of the 36 kindergarteners who began the study, three children did not participate in the 2 weeks post-training session. One child chose not to participate, one child exhibited reduced compliance, and one child was not available. One second grader and one adult could not attend the 24 h post-training session. The data from these participants were not included in the main analyses across the study period. However, their data were included in the correlation analyses that pertained to the training phase.

Two levels of analysis were performed. First, the overall production time and error rate were analyzed, in order to enable comparisons with other studies of skill learning that report speed and accuracy (Vinter and Perruchet, 2000; Dorfberger et al., 2007; Wilhelm et al., 2008; Savion-Lemieux et al., 2009). In light of the studies of drawing and handwriting by children (Adi-Japha and Freeman, 2001; Rosenblum et al., 2006), these were followed by analyses of the time spent while producing the shapes, the off-page time (i.e., while moving between the shapes and shape segments), the distance of the drawn line from the midpoint, and the number of strokes used to produce the shapes.

Performance was averaged across the blocks that constitute the four time points: initial training (blocks 1–4 on day 1, TP_1_); end-training (blocks 9–12 on day 1, TP_2_); 24 h post-training (four blocks, 24 h post-training, TP_3_); and 2 weeks post-training (four blocks, assessed 2 weeks after day 1, TP_4_). The data were subjected to a 3 (age group) × 4 (time points: initial training, end-training, 24 h post-training, and 2 weeks post-training) repeated measures ANOVA.

The results of the ANOVA analyses are presented in **Table 1**. **Table 1** further reports the analyses across the three study phases, defined as the difference between successive time points: training (= end-of-training – initial training; TP_2_–TP_1_ in **Table 1**), consolidation (= 24 h post-training – end-training; TP_3_–TP_2_)_,_ and retention (= 2 weeks post-training – 24 h post-training; TP_4_–TP_3_)_._

**Table 1:** Analysis of variance (ANOVA) results on the study production measures.

	DF overall	F- overall	DF phases	Training phase	Consolidation phase	Retention phase
**Production time**
Group	2, 68	82.03***	2, 68	61.18***	67.45***	78.05***
Time point	3, 204	53.06***	1, 68	63.21***	28.60***	0.33
Time point × Group	6, 204	4.73***	2, 68	7.67**	1.88	0.72
**Error rate**
Group	2, 68	9.24***	2, 68	4.42*	6.62**	8.88***
Time point	3, 204	1.13	1, 68	2.18	1.78	0.82
Time point × Group	6, 204	0.47	2, 68	0.98	0.35	0.07
**On-page production time**
Group	2, 68	55.46***	2, 68	39.51***	40.99***	52.72***
Time point	3, 204	34.96***	1, 68	62.42***	18.93***	0.00
Time point × Group	6, 204	2.78*	2, 68	6.81**	0.62	0.08
**Off-page production time**
Group	2, 68	72.72***	2, 68	52.52***	72.40***	73.48***
Time point	3, 204	34.04***	1, 68	33.64***	24.96***	2.03
Time point × Group	6, 204	4.48***	2, 68	4.74*	3.93*	6.30***
**Distance from midpoint**
Group	2, 68	2.88	2, 68	1.38	4.47*	3.63*
Time point	3, 204	7.57***	1, 68	0.11	3.36	5.75*
Time point × Group	6, 204	1.16	2, 68	2.12	0.01	0.22
**Number of pen strokes**
Group	2, 68	8.32**	2, 68	4.80*	6.26**	9.03***
Time point	3, 204	7.74***	1, 68	8.70**	0.01	0.11
Time point × Group	6, 204	0.95	2, 68	0.63	1.68	2.18

Overall analysis, and by study phases. *p < 0.05, **p < 0.01, ***p < 0.001.

### PRODUCTION TIMES

**FIGURE 2:**
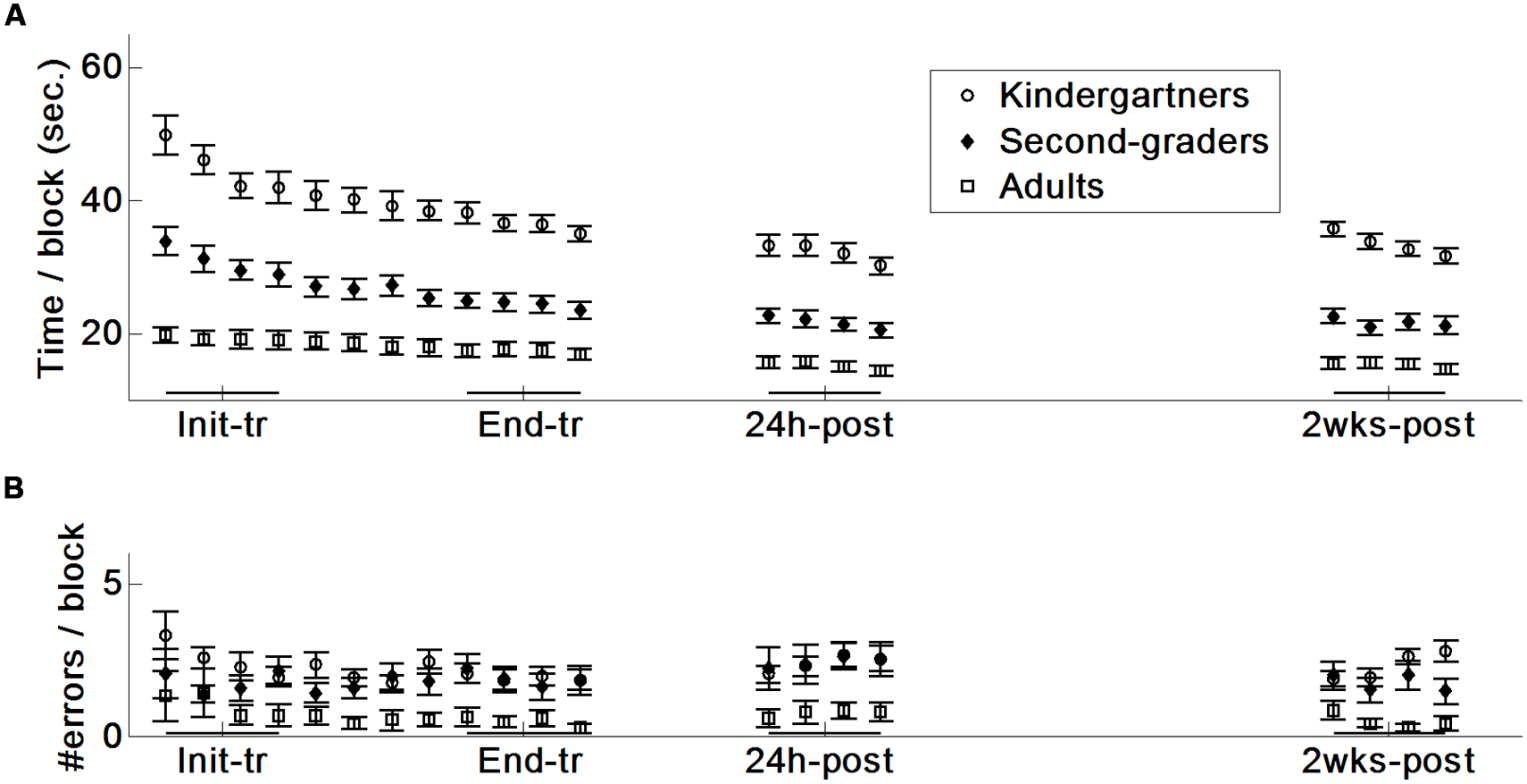
**Speed and accuracy data (mean and standard error): initial training (Init-tr, blocks 1–4 on day 1), end-training (End-tr, blocks 9–12 on day 1), 24 h post-training (24 h-post), and 2 weeks post-training (2 wks-post). (A)** Time per block. **(B)** Number of erroneous patterns produced.

The analysis of the production times indicated that the kindergarteners were slower than the second graders, who were slower than the adults (**Table 1** main effect of group followed by a Scheffé test, *p*s < 0.001; **Figure 2A**). However, all three groups improved their performance times across the four time points, albeit at a different rate, as indicated by a significant age group × time point interaction. This interaction was followed by dividing the analyses according to the three study phases: training, consolidation and retention. Significant group differences in the improvement rate emerged during the training phase. No differences in the improvement rate emerged during the consolidation (*p* = 0.16) or retention (*p* = 0.49) phase (**Table 1**).

#### Training

A follow-up analysis of the interaction during the training phase indicated that all three age groups improved their performance times. However, these gains were larger for the kindergarteners [*F*(1,50) = 13.45, *p* < 0.001, η^2^ = 0.21] and second graders [*F*(1,36) = 15.27, *p* < 0.001, η^2^ = 0.30] than for the adults. It should be noted that in all three age groups, the participants who were initially slower made larger gains during the training phase, as indicated by correlating initial performance with the reduction in performance time [*r*(36) = –0.86, *r*(20) = –0.65, and *r*(20) = –0.61, respectively by age, *p*s < 0.01]. This finding suggests that the larger improvement exhibited by the younger children may be related to their initial slow performance. However, age differences remained even relative to the initial performance level [*F*(2,68) = 7.67, *p* < 0.001, η^2^ = 0.18]: the kindergarteners and the second graders improved more than the adults (16 and 19 vs. 9%, respectively; Scheffé *p* < 0.001 and *p* < 0.05).

The three age groups did not differ in the slopes of their improvement across the last four blocks of the training day [*F*(2,68) = 1.52, *p* = 0.23], which was the basis for the comparison of performance improvement at 24 h post-training.

#### Consolidation

All three groups exhibited improvements in performance times during the consolidation phase (**Table 1**, consolidation phase, time point main effect; kindergarteners 29/33, second graders 17/19, and adults 18/19 showed improvement). A power-law analysis was used to further analyze performance gains by comparing the difference between the actual performances on the four blocks 24 h post-training to the expected performance. Expected performance was calculated by extrapolating the group average training curve to an additional four blocks. This extrapolation was based on a power-law function in the format “a^∗^X^-b^ + c”, which was fitted to the 12 training blocks (see Materials and Methods). The analysis indicated that the three groups improved beyond expectations based on their performance during training [*F*(1,68) = 11.53, *p* < 0.01, η^2^ = 0.12], with no difference between the age groups [*F*(2,68) = 0.25, *p* = 0.77]. The three age groups did not differ in their slope of improvement across the four blocks performed at 24 h post-training [*F*(2,68) = 0.70, *p* = 0.50].

#### Retention

No differences emerged in performance speed at 2 weeks compared with 24 h post-training, which indicates that performance was retained in all three age groups (*p* = 0.57). Furthermore, no differences emerged between the first block of the consolidation and retention phases in any of the age groups [*t*(32) = 0.28, *t*(19) = 0.36, and *t*(19) = 0.59 for the kindergarteners, second graders and adults, respectively, *p*s > 0.5]. However, analysis of the slopes of the four blocks tested at 2 weeks post-training indicated that the kindergarteners improved across these blocks more than the second graders and the adults [for the overall comparison, *F*(2,68) = 5.19, *p* < 0.01, η^2^ = 0.13; Scheffé, *p*s < 0.05].

### ERROR RATE

Analysis of error rates (i.e., the percentage of shapes that are too wide, too narrow or that were produced in more than one stroke) indicated that the kindergarteners and second graders made more errors than the adults (Scheffé, *p* < 0.001; **Table 1** and **Figure 2B**), with no difference between kindergartners and second graders. No further main effects or interactions emerged (see **Table 1**), suggesting that the three age groups maintained their high accuracy throughout the experiment, with an average accuracy of 85, 87 and 95%, for the kindergartners, second graders and adults, respectively. A within-group analysis further indicated that training and consolidation gains in production time did not occur at the expense of an increase in the error rate (*r*s < 0.26, *p*s > 0.13). This indicates the absence of a speed-accuracy tradeoff.

### ON-PAGE PRODUCTION TIME

**FIGURE 3:**
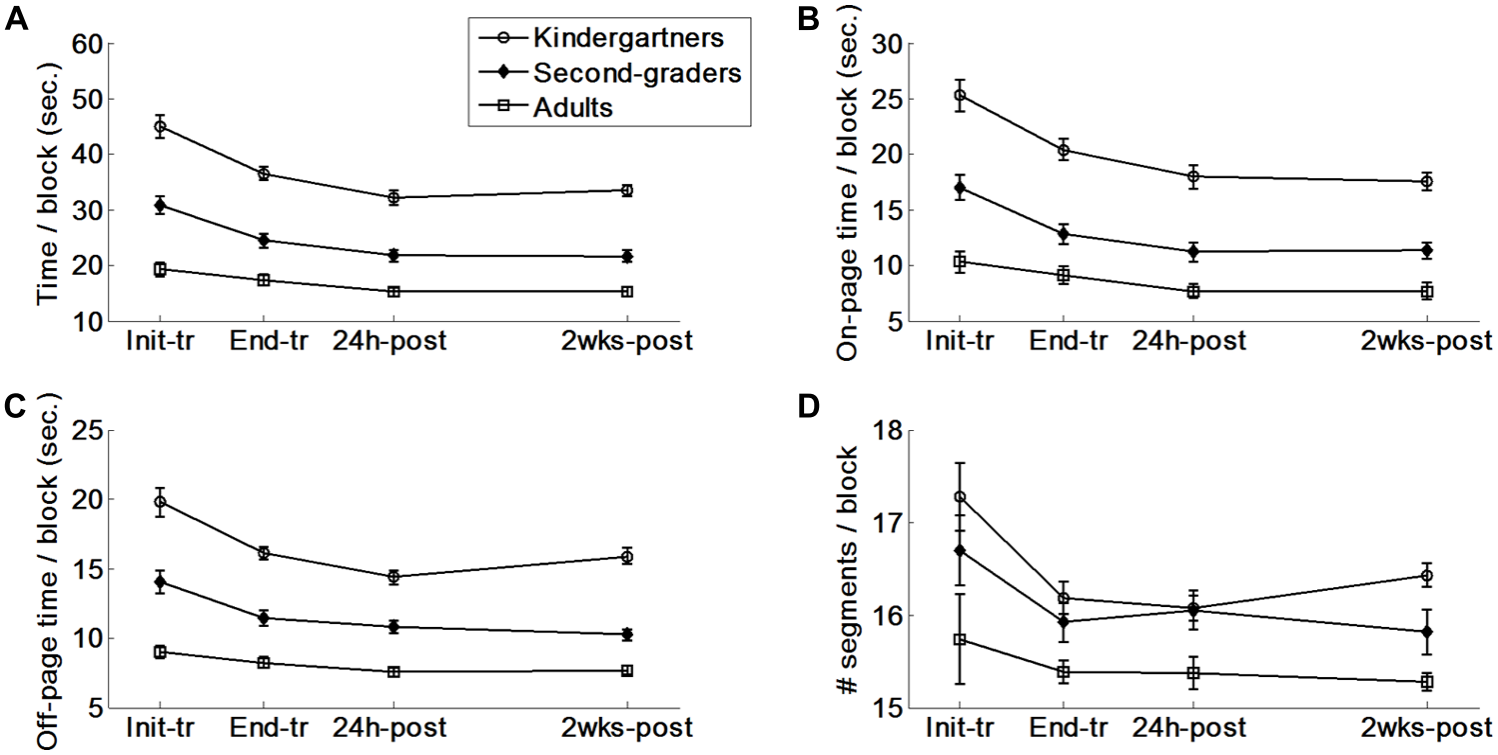
**Production data. (A)** Overall time. The four points represent the average across the four blocks of the four time points depicted in **Figure 2A**. **(B)** On-page time. **(C)** Off-page time. **(D)** Number of pen strokes per block. The minimum value is 15 strokes.

The task production time was further divided into two components (Rosenblum et al., 2003): the on-page and the off-page production times (**Figure 3A-C**). Analyses of the on-page time followed exactly the same pattern as the overall production time results, in which the kindergarteners had longer writing times than the second graders, who had longer writing times than the adults (Scheffé, *p*s < 0.01). Additionally, the 3 age groups reduced their on-page performance times across the four time points, but at a different rates, as indicated by a significant age group × time point interaction (**Table 1**).

A follow-up analysis on this interaction indicated that significant group differences in the improvement rates emerged only during the training phase. Although all three age groups improved their on-page performance times during the training phase, these gains were larger for the kindergarteners [*F*(1,50) = 12.39, *p* < 0.01, η^2^ = 0.20] and second graders [*F*(1,36) = 12.18, *p* < 0.001, η^2^ = 0.25] than for the adults.

All three groups improved their on-page performance times during the consolidation phase, with no differences between the groups in the gains accrued (*p* = 0.53). No differences emerged in performance speed at 2 weeks compared with 24 h post-training, indicating that performance was retained in all three age groups (*p* = 0.95).

### OFF-PAGE PRODUCTION TIME

The off-page time included the time spent when the pen was in the air while writing or moving between shapes or lines. The kindergarteners spent more time off-page than the second graders, who spent more time off-page than the adults (Scheffé, *p*s < 0.01). The 3 age groups reduced their off-page time across the four time points but at a different rate, as indicated by a significant age group × time point interaction. A follow-up analysis of this interaction indicated significant group differences in the rate of change during the training, consolidation, and retention phases.

During the training phase, the off-page time was reduced in all three age groups. The reduction was larger for the kindergarteners [*F*(1,50) = 7.90, *p* < 0.01, η^2^ = 0.14] and second graders [*F*(1,36) = 10.71, *p* < 0.01, η^2^ = 0.22] than for the adults. The off-page time continued to improve during the consolidation phase. The reduction in the off-page time was again larger for the kindergarteners [*F*(1,50) = 4.08, *p* < 0.05, η^2^ = 0.08] and second graders [*F*(1,50) = 4.05, *p* < 0.05, η^2^ = 0.08] than for the adults.

At the retention phase, significant group differences emerged in the performance level at 2 weeks compared with 24 h post-training between the kindergarteners and the second graders [*F*(1,50) = 8.36, *p* < 0.01, η^2^ = 0.14] and between the kindergarteners and the adults [*F*(1,50) = 4.65, *p* < 0.04, η^2^ = 0.09]. These differences emerged because the kindergarteners spent more time off-page during the retention testing than at 24 h post-training [*t*(32) = 3.07, *p* < 0.01], whereas the second graders and the adults did not exhibit this behavior [*t*s(18) < 1.22, *p*s > 0.23].

It should be noted that a larger decrease in the off-page time from the initial measurement to 24 h post-training was associated with a larger initial off-page time in all three age groups, as indicated by correlating off-page time at initial performance with the reduction in off-page time [*r*(33) = –0.89, *r*(19) = –0.85, and *r*(19) = –0.81, respectively by age, *p*s < 0.001]. This finding may explain why the kindergarteners improved more than the other two groups. After correcting for the initial performance (i.e., by comparing the improvement relative to the initial performance) this difference became non-significant [*F*(2,68) = 2.46, *p* = 0.093]. No correlation was found between the off-page time at 24 h post-training and the difference in the off-page time from 24 h to 2 weeks post-training (*r*s < 0.24, *p* > 0.18).

### DISTANCE FROM MIDPOINT

Overall, the distance of the drawn line from the midpoint (point “B” in **Figure 1A**) was similar across groups (**Table 1**, no main effect of group), although at the later phases the adults’ distance was larger than that of kindergarteners (significant differences at the end of the training and at 24 h post-training, Scheffé *p* < 0.05). With practice, the distance increased. The increase in distance from the midpoint became significant during the retention phase, indicating that the distance was significantly larger at 2 weeks post-training than 24 h post-training (at 24 h post-training the distance was *M* = 1.28 mm, SD = 0.34, at 2 weeks post-training *M* = 1.38 mm, SD = 0.43, both well within the allowed distance of up to 2.75 mm from the midpoint). This increase was accompanied by a decrease in the width of the produced shapes [*F*(1,68) = 4.71, *p* < 0.04, η^2^ = 0.07, no age group difference], due to the tendency to draw the line as going on the left border of the mid-point circle (about 1.5 mm from the midpoint).

### NUMBER OF PEN STROKES

Overall, the kindergarteners and second graders used more pen strokes than the adults to produce the desired shapes (Scheffé, *p*s < 0.01; **Figure 3D**). The participants in all 3 age groups reduced the number of pen strokes across the four time points. These reductions occurred primarily during the training phase. No further changes were observed during the consolidation or retention phases (**Table 1**).

Because of the association between the off-page time and the number of strokes reported in the literature for children with low graphic skills (Rosenblum et al., 2003, 2006), we analyzed the change in the number of pen strokes for each group during the retention phase (i.e., from 24 h to 2 weeks post-training). This number significantly increased only for the kindergarteners (from 15.54 to 15.78 strokes per block, on average, SE = 0.10 strokes; 15 is the minimal number of pen strokes). It should be noted that although the increase in pen strokes was statistically significant, it has no practical significance (an increase of 0.24 strokes per block on average per child).

### ASSOCIATIONS BETWEEN LEARNING AND SEQUENTIAL SHORT-TERM MEMORY

To test the role of short term memory, correlation analyses were performed between the two sequential short-term memory tests (i.e., the Number Recall test and the Hand Movement test) and the performance measures that showed change: the gains accrued during the training and consolidation phases (in on-page and off-page time), as well as losses during retention (air-time and number-of segments in kindergarteners). None of the correlations were significant at the *p* < 0.01 level. However, low correlations were observed for kindergarten children between the Hand Movement test and two performance measures: the reduction in the on-page time during the training phase [*r*(36) = –0.34, *p* < 0.05], and the increase in the number of segments in the retention phase [*r*(33) = –0.38, *p* < 0.03].

## DISCUSSION

In the current study, 5- to 6-years-old kindergarteners, 7- to 8-years-old second graders and young adults learned to produce a grapho-motor pattern. Our results indicate that training on the graphic symbol task resulted in significant gains concurrent with the training experience in performance time in all three age groups. Larger gains occurred for younger participants and individuals who initially performed more poorly. Between-session gains, as expressed at 24 h after the termination of the training session, were also exhibited by the three age groups. Gains in production time did not occur at the expense of an increase in the error rate, which was maintained. This demonstrates that major characteristics of skill acquisition, previously defined in adults (Censor et al., 2012), are typical of children’s learning. Our findings are similar to previous results of a developmental skill acquisition study in 9-years-old children and adults that used a more complex task (Dorfberger et al., 2007). Effective skill learning in young children can therefore be studied using this simple daily task, which does not require a great deal of attentional resources or declarative elements. Nevertheless, our findings suggest that an age advantage may exist in the long-term retention of skills.

Various studies of skill learning that compared children with young adults have reported differences in performance gains accrued during a given training experience. In different studies, either the adults (Thomas et al., 2004; Huyck and Wright, 2011; Vasudevan et al., 2011; Lejeune et al., 2013) or the children (Fischer et al., 2007; Bishop et al., 2012; Janacsek et al., 2012) exhibit an advantage. The results have differed even for the same task under different training lengths (Thomas et al., 2004; Fischer et al., 2007), and may depend on the method of comparing age groups (Janacsek et al., 2012), with adults showing better learning in shorter training lengths and in terms of normalized gains. Advantages in retention may further depend on the interval of delay studied, with children showing an advantageous retention after a delay of a few hours as opposed to days (Bishop et al., 2012; Ashtamker and Karni, 2013). Another line of research suggests that procedural-implicit skill learning is age-invariant (Reber, 1993; Meulemans et al., 1998; Vinter and Perruchet, 2000; Karatekin et al., 2007). Recent studies have attempted to address the disparity in previous findings (Lejeune et al., 2013; Nemeth et al., 2013; Witt et al., 2013; Lukacs and Kemeny, 2014). These authors suggest an additional account by which skill learning *per se* is effective at young ages. However, participants in different age groups use different strategies. For example, older participants rely more on executive attention control, sensorimotor integration, and the ability to build complex internal models and representational units (Ferrel-Chapus et al., 2002; Nemeth et al., 2013; Witt et al., 2013; Hodel et al., 2014). As a result, young adults have an apparent advantage over children in skill learning. The findings of the current study indicated higher training gains in younger children. This finding remained after improvement was considered proportionally to the younger children’s lower initial performance. Our data thus support the notion of childhood advantage or the assumption of age-invariance in gains accrued during the training of a novel task. Our data *do not* indicate qualitative differences in temporal or spatial features of the task acquisition between age groups. It should be noted, however, that skill learning studies have focused primarily on tasks that are acquired implicitly (e.g., statistical learning), whereas the current task was acquired explicitly (procedural-explicit learning, Robertson et al., 2004). Furthermore, from an educational point of view, our observation that children with a lower initial performance speed improved more than their peers underscores the benefits of training in the improvement of procedural skills (e.g., handwriting proficiency) and potentially other academic skills that can benefit from repeated practice (e.g., reading).

Age-related differences emerged in retention testing 2 weeks post-practice day. Although the overall level of performance was retained in all three age groups, a more detailed analysis revealed that the kindergarteners spent more time off-page and used slightly more segments for producing the shapes on the retention testing compared with the 24 h post-training assessment. Longer off-page periods and a more extensive use of pen strokes characterize the writing of children with low grapho-motor skills (Rosenblum et al., 2006) or low-attention (Adi-Japha et al., 2007). The study of handwriting skills suggests that the off-page time is associated not only with a move between writing elements but also with the planning of the next writing segment (Rosenblum et al., 2003, 2006). The number of strokes in grapho-motor production is a feature of fluent movement. In the current study, it is also a feature of accuracy. Accuracy is considered to be related to attention control and declarative task elements (Savion-Lemieux and Penhune, 2005; Janacsek et al., 2012). Thus, it may be suggested that with the passage of time, kindergarteners find it more difficult to retrieve the more explicit-, attentive-, or planning-related task elements that affect the shape of the trajectory. This is in contrast to lower-level motor execution processes that are expressed in the mean duration of shape performance (Sosnik et al., 2014) and were well-retained. A difficulty in retrieval of explicit task elements following an 8-weeks retention period has been identified in adults (Savion-Lemieux and Penhune, 2005). This finding may suggest that these less efficient retrieval behaviors are not unique to children. The finding of an age advantage during retention testing is consistent with a previous study by Dorfberger et al. (2007), who reported that adults exhibited improved performance on a finger-to-thumb opposition movement sequence task following 6 weeks of retention, whereas children maintained their performance level.

Previous developmental studies of skill learning have not identified associations between short-term or working memory performance assessed with the digit-span tasks and measures of learning (Savion-Lemieux et al., 2009; Lejeune et al., 2013). In the current study, however, kindergarteners’ changes in performance were associated with a motor measure of sequential short-term memory. This difference may have occurred because the current study used a task-specific (i.e., visual-motor) measure. A lower short-term motor memory span was associated with less on-page time improvement during training, and with an increase in the number of segments used on retention testing. Theories of skill learning, as well as imaging studies in adults, suggest that the fast learning phase (i.e., the training phase before a performance plateau is reached) is characterized by processes such as trial and error and the adaptation of performance solutions, as well as a more controlled execution in general (Anderson, 1982; Logan, 1988; Chein and Schneider, 2005; Doyon and Benali, 2005). These processes are related to interactions between the motor and pre-motor cortical regions and the prefrontal regions of the brain (Dayan and Cohen, 2011). The later phases of learning (i.e., consolidation or later learning) are characterized by less involvement of attentional and executive resources, and therefore correlation with short-term memory is not expected. The finding that kindergarten children with lower sequential short-term motor memory increased the number of segments used at retention testing support the hypothesis that this increase is related to planning and attentive task elements. Associations with short-term memory were restricted to kindergarteners, possibly because for second-graders the task was easy to perform and retain. As in previous studies (Savion-Lemieux et al., 2009), the verbal sequential short-term memory test was not associated with measures of learning. Digit-span tasks are considered short-term verbal/phonological memory tests (e.g., Gray, 2006) and are therefore less related to motor skill acquisition than motor sequential tasks.

Although the adults outperformed the kindergartners and second graders in terms of accuracy, the accuracy levels were maintained throughout the task in all age groups. Interestingly, the kindergarteners and second graders had a similar number of errors. However, the second graders were faster than the kindergarteners. These data suggest a different developmental pattern for speed and accuracy and are consistent with findings of other developmental studies of skill acquisition that used different paradigms (Savion-Lemieux et al., 2009; Janacsek et al., 2012). In addition, these findings strengthen the model proposed by Hikosaka et al. (2002), that motor skills are acquired and retained in two independent but parallel forms: speed and accuracy.

When considering the conclusions of this study, it should be taken into account that this is a small-scale study, and other developmental factors not examined in this study (e.g., attention) may have affected the results. The current study focused on younger children. However, more age groups should be tested in order to develop a comprehensive developmental understanding. Nevertheless, the current study extends the findings of previous researches which suggest that the stages of skill acquisition observed in adults are present not only in school-aged children but also in early childhood. These stages are present despite the numerous developmental differences in learning-related skills, such as memory span, working memory, attention, and planning. While the current study supports several associations between sequential motor short-term memory and sequential motor learning, more elaborate studies should be conducted to examine whether and how basic cognitive functions interact with skill learning (e.g., Fox et al., 2014).

## Conflict of Interest Statement

The authors declare that the research was conducted in the absence of any commercial or financial relationships that could be construed as a potential conflict of interest.

## References

[B1] Adi-Japha E. (2013). “The assessment of skill learning in African children,” in *Neuropsychology of Children in Africa*, eds Boivin M. J. Giordani B. (New York: Springer) 215–234 10.1007/978-1-4614-6834-9_11

[B2] Adi-Japha E. Abu-Asba H. (2014). Learning, forgetting, and relearning: skill learning in children with language impairment. *Am. J. Speech Lang. Pathol.* 23 696–707 10.1044/2014_AJSLP-13-0031 25215440

[B3] Adi-Japha E. Badeer R. Dorfberger S. Karni A. (2014). Rapid motor memory stabilization in childhood. *Dev. Sci.* 17 424–433 10.1111/desc.12132 24620995

[B4] Adi-Japha E. Freeman N. H. (2001). Development of differentiation between writing and drawing systems. *Dev. Psychol.* 37 101–114 10.1037/0012-1649.37.1.101 11206425

[B5] Adi-Japha E. Landau Y. Frenkel L. Teicher M. Gross-Tzur V. Shalev R. S. (2007). ADHD and dysgraphia: underlying mechanisms. *Cortex* 43 700–709 10.1016/S0010-9452(08)70499-4 17710822

[B6] Adi-Japha E. Strulovich-Schwartz O. Julius M. (2011). Delayed motor skill acquisition in children with language impairment. *Res. Dev. Disabil.* 32 2963–2971 10.1016/j.ridd.2011.05.005 21624816

[B7] Anderson J. R. (1982). Acquisition of cognitive skill. *Psychol. Rev.* 89 369–406 10.1037/0033-295X.89.4.369

[B8] Ashtamker L. Karni A. (2013). Motor memory in childhood: early expression of consolidation phase gains. *Neurobiol. Learn. Mem.* 106 26–30 10.1016/j.nlm.2013.07.003 23867636

[B9] Beery K. E. Buktenica N. A. Berry N. A. (1997). *The Beery-Buktenica Developmental Test of Visual-Motor Integration: VMI with Supplemental Developmental Tests of Visual Perception and Motor Co-ordination: Administration, Scoring and Teaching Manual,* 4th Edn. Parsippany, NJ: Modern Curriculum Press.

[B10] Bishop D. V. Barry J. G. Hardiman M. J. (2012). Delayed retention of new word-forms is better in children than adults regardless of language ability: a factorial two-way study. *PLoS ONE* 7:e37326 10.1371/journal.pone.0037326 PMC3353950 22615979

[B11] Brown R. M. Robertson E. M. (2007). Off-line processing: reciprocal interactions between declarative and procedural memories. *J. Neurosci.* 27 10468–10475 10.1523/JNEUROSCI.2799-07.2007 17898218 PMC6673170

[B12] Cameron C. E. Brock L. L. Murrah W. M. Bell L. H. Worzalla S. L. Grissmer D. (2012). Fine motor skills and executive function both contribute to kindergarten achievement. *Child Dev.* 83 1229–1244 10.1111/j.1467-8624.2012.01768.x 22537276 PMC3399936

[B13] Caroni P. Donato F. Muller D. (2012). Structural plasticity upon learning: regulation and functions. *Nat. Rev. Neurosci.* 13 478–490 10.1038/nrn3258 22714019

[B14] Censor N. Sagi D. Cohen L. G. (2012). Common mechanisms of human perceptual and motor learning. *Nat. Rev. Neurosci.* 13 658–664 10.1038/nrn3315 22903222 PMC4880370

[B15] Chein J. M. Schneider W. (2005). Neuroimaging studies of practice related change: fMRI and meta-analytic evidence of a domain-general control network for learning. *Cogn. Brain Res.* 25 607–623 10.1016/j.cogbrainres.2005.08.013 16242923

[B16] Cohen M. J. (1997). *Manual for the Children’s Memory Scale*. San Antonio, TX: The Psychological Association.

[B17] Cohen N. J. Squire L. R. (1980). Preserved learning and retention of pattern analyzing skill in amnesia: dissociation of knowing how and knowing that. *Science* 210 207–209 10.1126/science.7414331 7414331

[B18] Dayan E. Cohen L. G. (2011). Neuroplasticity subserving motor skill learning. *Neuron* 72 443–454 10.1016/j.neuron.2011.10.008 22078504 PMC3217208

[B19] Dorfberger S. Adi-japha E. Karni A. (2007). Reduced susceptibility to interference in the consolidation of motor memory before adolescence. *PLoS ONE* 2:e240 10.1371/journal.pone.0000240 PMC1800346 17327907

[B20] Doyon J. Benali H. (2005). Reorganization and plasticity in the adult brain during learning of motor skills. *Curr. Opin. Neurobiol.* 15 161–167 10.1016/j.conb.2005.03.004 15831397

[B21] Feldman D. E. (2009). Synaptic mechanisms for plasticity in neocortex. *Ann. Rev. Neurosci.* 32 33–55 10.1146/annurev.neuro.051508.135516 19400721 PMC3071739

[B22] Ferrel-Chapus C. Hay L. Olivier I. Bard C. Fleury M. (2002). Visuomanual coordination in childhood: adaptation to visual distortion. *Exp. Brain Res.* 144 506–517 10.1007/s00221-002-1064-2 12037635

[B23] Fischer S. Wilhelm I. Born J. (2007). Developmental differences in sleep’s role for implicit off-line learning: comparing children with adults. *J. Cogn. Neurosci.* 19 214–227 10.1162/jocn.2007.19.2.214 17280511

[B24] Fox O. Adi-Japha E. Karni A. (2014). A skipped dose (placebo) of methylphenidate can reduce motor skill acquisition but not retention in adolescents with Attention Deficit Hyperactivity Disorder. *Eur. Neuropsychopharmacol.* 24 391–396 10.1016/j.euroneuro.2013.11.005 24332892

[B25] Gray S. (2006). The relationship between phonological memory, receptive vocabulary, and fast mapping in young children with specific language impairment. *J. Speech Lang. Hear. Res.* 49 955–969 10.1044/1092-4388(2006/069) 17077208

[B26] Grissmer D. Grimm K. J. Aiyer S. M. Murrah W. M. Steele J. S. (2010). Fine motor skills and early comprehension of the world: two new school readiness indicators. *Dev. Psychol.* 46 1008–1017 10.1037/a0020104 20822219

[B27] Hauptmann B. Reinhart E. Brandt S. A. Karni A. (2005). The predictive value of the leveling off of within-session performance for procedural memory consolidation. *Cogn. Brain Res.* 24 181–189 10.1016/j.cogbrainres.2005.01.012 15993756

[B28] Hikosaka O. Nakamura K. Sakai K. Nakahara H. (2002). Central mechanisms of motor skill learning. *Curr. Opin. Neurobiol.* 12 217–222 10.1016/S0959-4388(02)00307-0 12015240

[B29] Hodel A. S. Markant J. C. Van Den Heuvel S. E. Cirilli-Raether J. M. Thomas K. M. (2014). Developmental differences in effects of task pacing on implicit sequence learning. *Front. Psychol.* 5:153 10.3389/fpsyg.2014.00153 PMC3934418 24616712

[B30] Huyck J. J. Wright B. A. (2011). Late maturation of auditory perceptual learning. *Dev. Sci.* 14 614–621 10.1111/j.1467-7687.2010.01009.x 21477199 PMC3107547

[B31] Janacsek K. Fiser J. Nemeth D. (2012). The best time to acquire new skills: age-related differences in implicit sequence learning across the human lifespan. *Dev. Sci.* 15 596–505 10.1111/j.1467-7687.2012.01150.x PMC3383816 22709399

[B32] Karatekin C. Marcus D. J. White T. J. (2007). Oculomotor and manual indices of incidental and intentional spatial sequence learning in middle childhood and adolescence. *J. Exp. Child Psychol.* 96 107–130 10.1016/j.jecp.2006.05.005 16828110

[B33] Kaufman A. S. Kaufman N. L. (1983). *Kaufman Assessment Battery for Children, K-AB. Administration and Scoring Manual*. Circle Pines, MN: American Guidance Service Inc.

[B34] Kaufman A. S. Kaufman N. L. (2004). *Manual for the Kaufman Assessment Battery for Children,* 2nd Edn. Circle Pines, MN: AGS Publishing.

[B35] Korman M. Raz N. Flash T. Karni A. (2003). Multiple shifts in the representation of motor sequence during the acquisition of skilled performance. *Proc. Natl. Acad. Sci. U.S.A.* 100 12492–12497 10.1073/pnas.2035019100 14530407 PMC218785

[B36] Lejeune C. Catale C. Schmitz X. Quertemont E. Meulemans T. (2013). Age-related differences in perceptuomotor procedural learning in children. *J. Exp. Child Psychol.* 116 157–168 10.1016/j.jecp.2013.05.001 23773917

[B37] Logan G. D. (1988). Toward an instance theory of automatization. *Psychol. Rev.* 95 492–527 10.1037/0033-295X.95.4.492

[B38] Longcamp M. Zerbato-Poudou M. T. Velay J. L. (2005). The influence of writing practice on letter recognition in preschool children: a comparison between handwriting and typing. *Acta Psychol.* 119 67–79 10.1016/j.actpsy.2004.10.019 15823243

[B39] Lukacs A. Kemeny F. (2014) Development of different forms of skill learning throughout the lifespan. *Cogn. Sci.* 10.1111/cogs.12143 [Epub ahead of print] 25039658

[B40] Meisels S. J. Marsden D. B. Wiske M. S. Henderson L. W. (1997) *Early Screening Inventory-Revised* New York, NY: Pearson Early Learning.

[B41] Meulemans T. Van der Linden M. Perruchet P. (1998). Implicit sequence learning in children. *J. Exp. Child Psychol.* 69 199–221 10.1006/jecp.1998.2442 9654439

[B42] Nemeth D. Janacsek K. Fiser J. (2013). Age-dependent and coordinated shift in performance between implicit and explicit skill learning. *Front. Comput. Neurosci.* 7:147 10.3389/fncom.2013.00147 PMC3805033 24155717

[B43] Oldfield R. C. (1971). The assessment and analysis of handedness: the Edinburgh inventory. *Neuropsychologia* 9 97–113 10.1016/0028-3932(71)90067-4 5146491

[B44] Phizer M. Shimborsky G. Walf N. Hazani A. (1995). *The Kaufman Assessment Battery for Children-Israeli Version.* Jerusalem: Henrietta Szold Institute.

[B45] Reber A. S. (1993). *Implicit Learning and Tacit Knowledge: An Essay on the Cognitive Unconscious*. New York: Oxford University Press.

[B46] Rickard T. C. (2007). Forgetting and learning potentiation: dual consequences of between-session delays in cognitive skill learning. *J. Exp. Psychol. Learn. Mem. Cogn.* 33 297–304 10.1037/0278-7393.33.2.297 17352612

[B47] Robertson E. M. Pascual-Leone A. Miall R. C. (2004). Current concepts in procedural consolidation. *Nat. Rev. Neurosci.* 5 576–582 10.1038/nrn1426 15208699

[B48] Rosenblum S. Dvorkin A. Y. Weiss P. L. (2006). Automatic segmentation as a tool for examining the handwriting process of children with dysgraphic and proficient handwriting. *Hum. Mov. Sci.* 25 608–621 10.1016/j.humov.2006.07.005 17011656

[B49] Rosenblum S. Weiss P. L. Parush S. (2003). Product and process evaluation of handwriting difficulties. *Educ. Psychol. Rev.* 15 41–81 10.1023/A:1021371425220

[B50] Savion-Lemieux T. Bailey J. A. Penhune V. (2009). Developmental contributions to motor sequence learning. *Exp. Brain Res.* 195 293–306 10.1007/s00221-009-1786-5 19363605

[B51] Savion-Lemieux T. Penhune V. (2005). The effects of practice and delay on motor skill learning and retention. *Exp. Brain Res.* 161 423–431 10.1007/s00221-004-2085-9 15551084

[B52] Scheffé H. (1959). *The Analysis of Variance.* New York: Wiley (Reprinted 1999, ISBN 0-471-34505-9)

[B53] Schendel J. D. Shields J. C. Katz M. C. (1978) *Retention of Motor Skill: Review (Technical Paper 113)* Alexandria, VA: U.S. Army Research Institute for the Behavioral and Social Sciences.

[B54] Sosnik R. Flash T. Sterkin A. Hauptmann B. Karni A. (2014). The activity in the contralateral primary motor cortex, dorsal premotor and supplementary motor area is modulated by performance gains. *Front. Neurosci.* 8:201 10.3389/fnhum.2014.00201 PMC3997032 24795591

[B55] Sosnik R. Hauptman B. Karni A. Flash T. (2004). When practice leads to co-articulation: the evolution of geometrically defined movement primitives. *Exp. Brain Res.* 156 422–438 10.1007/s00221-003-1799-4 15167977

[B56] Sosnik R. Karni A. Flash T. (2007). The acquisition and implementation of the smoothness maximization motion strategy is dependent on spatial accuracy demands. *Exp. Brain Res.* 176 311–331 10.1007/s00221-006-0617-1 16874514

[B57] Sumner E. Connelly V. Barnett A. L. (2014). The influence of spelling ability on handwriting production: children with and without dyslexia. *J. Exp. Psychol. Learn. Mem. Cogn.* 40 1441–1447 10.1037/a0035785 24548322

[B58] Thomas K. M. Hunt R. H. Vizueta N. Sommer T. Durston S. Yang Y. (2004). Evidence of developmental differences in implicit sequence learning: an fMRI study of children and adults. *J. Cogn. Neurosci.* 16 1339–1351 10.1162/0898929042304688 15509382

[B59] Vasudevan E. V. Torres-Oviedo G. Morton S. M. Yang J. F. Bastian A. J. (2011). Younger is not always better: development of locomotor adaptation from childhood to adulthood. *J. Neurosci.* 31 3055–3065 10.1523/JNEUROSCI.5781-10.2011 21414926 PMC3084584

[B60] Vinter A. Perruchet P. (2000). Implicit learning in children is not related to age: evidence from drawing behavior. *Child Dev* 71 1223–1240 10.1111/1467-8624.00225 11108093

[B61] Wilhelm I. Fischer S. Born J. (2008). Sleep in children improves memory performance on declarative but not procedural tasks. *Learn. Mem.* 15 373–377 10.1101/lm.803708 18441295

[B62] Witt A. Puspitawati I. Vinter A. (2013). How explicit and implicit test instructions in an implicit learning task affect performance. *PLoS ONE* 8:e53296 10.1371/journal.pone.0053296 PMC3541178 23326409

[B63] Xu T. Yu X. Perlik A. J. Tobin W. F. Zweig J. A. Tennant K. (2009). Rapid formation and selective stabilization of synapses for enduring motor memories. *Nature* 462 915–919 10.1038/nature08389 19946267 PMC2844762

